# Human hepatocyte carcinogenesis

**DOI:** 10.3892/ijo.2013.1829

**Published:** 2013-02-19

**Authors:** HIDENORI SHIRAHA, KAZUHIDE YAMAMOTO, MASAYOSHI NAMBA

**Affiliations:** 1 Department of Gastroenterology and Hepatology, Okayama University Faculty of Medicine, Okayama 700-8558;; 2 Niimi College, Nishikata 1263-2, Niimi 718-8585, Japan

**Keywords:** hepatocarcinogenesis, tumor suppressor genes, gene alteration

## Abstract

Hepatocellular carcinoma is the third most frequent cause of cancer-related death worldwide; and its incidence rate is increasing. Clinical and molecular medical analyses have revealed substantial information on hepatocarcinogenesis. Hepatocarcinogenesis is a stepwise process during which multiple genes are altered. Genetic changes and their biological consequences in human HCC can be divided into at least 4 groups: i) tumor suppressor genes (p53, retinoblastoma, phosphatase tensin homolog and runt-related transcription factor 3), ii) oncogenes (myc, K-ras, BRAF), iii) reactivation of developmental pathways (Wnt, hedgehog), and iv) growth factors and their receptors (transforming growth factor-α, insulin-like growth factor-2 receptor). An experimental model of human hepatocarcinogenesis such as *in vitro* neoplastic transformation of human hepatocytes has not been successfully achieved yet, but several immortalized human hepatocyte cell lines have been established. These immortalized human hepatocytes will become useful tools for the elucidation of hepatocarcinogenesis, especially for the initial step of multistep hepatocarcinogenesis.

## Contents

1. General aspects of hepatocellular carcinoma
2. Molecular alterations in human HCC
3. Experimental hepatocarcinogenesis *in vitro*
4. Experimental animal model of hepatocarcinogenesis
5. Conclusion

## 1. General aspects of hepatocellular carcinoma

Hepatocellular carcinoma (HCC) is the sixth most common cancer and third most frequent cause of cancer-related death worldwide (1–3). It has received considerable attention in recent years because of its rapid increase in incidence. Most HCC cases occur in sub-Saharan Africa and Eastern Asia. However, the incidence has been increasing in some developed countries including Japan, UK, France, and USA (1).

Chronic viral infection with the hepatitis B virus (HBV) or hepatitis C virus (HCV) appears to be the most significant causes of HCC (4). Chronic inflammation and regeneration of hepatocytes are underlying causes of HCC. Continuous inflammation occasionally damages DNA in the hepatocytes of the regenerating liver, thereby increasing the chances of gene alteration related to carcinogenesis.

Patients diagnosed with HCC have a poor prognosis because of the aggressive nature of the disease (1,5). Surgical resection or local ablation therapy is effective only at an early stage of HCC. However, approximately 70% of these patients develop recurrent tumors within 5 years (6). Moreover, no effective chemotherapy exists for the advanced disease. Molecular target therapy, especially that targeting the angiogenesis pathway, is now developing as a novel anti-cancer modality (7,8). This therapy seems to be a promising way of prolonging the survival of patients with advanced HCC.

Elucidation of the mechanism of hepatocarcinogenesis should contribute to the development of molecular target therapy. Although there is a growing understanding of the molecular mechanisms that induce hepatocarcinogenesis, real mechanisms of hepatocarcinogenesis have not been completely elucidated. However, cumulative knowledge regarding the molecular mechanisms of carcinogenesis revealed that the development and progression of HCC are caused by the accumulation of genetic changes, thus resulting in altered expression of cancer-related genes.

## 2. Molecular alterations in human HCC

### p53

The p53 gene is the most extensively studied gene in the solid tumors. Mutation of this gene has been identified in a variety of human cancers (9–12). The p53 pathways have many crucial roles in cell cycle control, transcriptional regulation, and apoptosis (13,14). Alteration of the p53 gene occurs at a relatively low frequency in HCC compared to other solid tumors. Epidemiologically p53 mutation was frequently found in aflatoxin-induced HCC (∼50%), but was rare in HCC that was not induced by aflatoxin (28–42%) (15–18). In a study of hepatitis B and C, the p53 mutation profile was different for both; the p53 abnormality in HBV-related HCC (45%) was significantly higher than that in HCV-related HCC (13%) (19). HBX protein, encoded by HBV genome, has been reported to be a transcriptional transactivator protein. In a transgenic model, HBX protein induced progressive neoplastic changes in the liver (20). This protein binds to the p53 protein in the cytoplasm, resulting in the blockage of p53 entry into the nucleus.

### Rb/p16

The retinoblastoma (Rb) gene is another widely studied tumor suppressor gene in HCC and other solid tumors. It is a negative regulator of the cell cycle through its ability to bind the transcription factor E2F and to suppress the transcription of S-phase-related genes (21,22). Mutations of Rb were found in only 15% of HCC cases (15). However, the loss of heterozygosity (LOH) of 13q, where the Rb gene is located, occurred more frequently in HCC (25–48%) (23,24).

The p16 gene, also known as cyclin-dependent kinase inhibitor 2A gene, is the regulator of the Rb pathway. Inactivation of either Rb or p16 was frequently found in HCC (81%) (25). Alterations of the p16 gene occurred either by promoter hyper-methylation or LOH of 9p in HCC (26,27).

### PTEN

Phosphatase and tensin homolog (PTEN) is a tumor suppressor gene located on chromosome 10q. It negatively regulates the phosphoinositide 3-kinase/Akt signaling pathway, which is involved in the regulation of cell survival (28). Absence or reduced expression of PTEN was found in ∼40% of HCC cases (29).

### RUNX3

Runt-related transcription factor 3 (RUNX3), located in chromosome 1p36, was first reported as a tumor suppressor gene for gastric cancer (30). RUNX3 is a potential tumor suppressor gene for HCC, as the decreased mRNA expression of RUNX3 was observed in 50–92% of HCC cases (31,32). The significance of decreased expression of RUNX3 is related to dysfunction of cell cycle regulation, decrement of apoptosis (33,34), enhancement of angiogenesis, and the development of epithelial-mesenchymal transition (35).

Most of gene alterations in tumor suppressor genes are due to LOH or promoter hypermethylation (36–39). Highest percentages of LOH were detected at several losi on chromosomes 8p, 4q, 4q, 17p, 16q, 6q, 1p and 9p in HCC (18). LOH of 17p and 9p are correlated with p53 and p16, respectively.

### Oncogenes

The role of the oncogenes in HCC seems to be less important as compared to that of the tumor suppressor genes, in contrast to other types of cancer. Myc, located on chromosome 8q, is a potent proto-oncogene in HCC and other cancers. It codes for a protein involved in nucleic acid metabolism and in mediating the cellular response to growth factors. The correlation of myc expression and tumor size was reported (40). Inactivation of myc suppressed the progression of HCC in a mouse model (41).

Mutation of the 3 major ras proto-oncogenes (H-, K-, and N-ras) was found in only in few cases of HCCs (42–44). K-ras mutation was frequently found in vinyl chloride related HCC (45). Activating point mutations of the BRAF gene occurred in 14% of HCC cases (46).

### Reactivation of developmental pathways

The Wnt/β-catenin pathway plays an essential role in liver development. Activation of the catenin pathway frequently occurred in HCC (47). The gene related to adenomatous polyposis coli (APC), a crucial regulator of intestinal carcinogenesis, is also involved in hepatocarcinogenesis. APC expression was reduced in HCC (48). This reduction induces the activation of the β-catenin signaling pathway. Mutation of β-catenin was also observed in HCC (49); mutation of this pathway contributes to the activation of the Wnt signaling pathway.

Hedgehog signaling is another developmental pathway that is involved in hepatocarcinogenesis (50). Hedgehog plays an important role in early embryonic development (51). Sonic hedgehog, Indian hedgehog, and desert hedgehog are 3 mammalian hedgehog genes that have been identified. Two major groups of hedgehog-related proteins that have been identified are patched (PTCH) and smoothened (SMO) (52–54). These two molecules interact with each other. In the absence of ligand, PTCH inhibits SMO. When hedgehog reaches the PTCH receptor, it binds to PTCH and releases the repression of SMO. Gli proteins, which are downstream signaling molecules of SMO, act as transcription factors, thus resulting in the promotion of cell growth and inhibition of apoptosis (55). The transcription of hedgehog and related molecules was reported to be increased in some cases of HCC (56).

### Growth factors and their receptors

The expression of several growth factors has been reported in HCC. Expression of the transforming growth factor-α (TGF-α) was increased in most cases of HCCs (81%) (57). TGF-α stimulates the proliferation of HCC cells by activating the epidermal growth factor receptor signaling pathway. Overexpression of TGF-α might be associated with hepatitis B infection (58).

The insulin-like growth factor-2 (IGF-2) signaling pathway is also involved in hepatocarcinogenesis. LOH or mutation of the IGF-2 receptor was frequently found (25–55%) in HCC (59,60). Alteration of this receptor is related to the overexpression of mitogen IGF-2, because the receptor induces the degradation of IGF-2. The IGF-2 receptor also activates transforming growth factor-β (TGF-β), a negative regulator of cell growth, by binding to the latent complex of TGF-β (61). Alteration of the TGF-β receptor type II gene itself was also found in HCC (∼10%) (62).

### Telomerase activity and telomere length

Telomere is a region of repetitive DNA at the end of each chromosome, which contributes to the stability and integrity of the chromosome (63). The length of the telomere is maintained by the activity of telomerase, which is a ribonucleoprotein complex composed of telomerase reverse transcriptase (TERT) and an RNA primer sequence. Without TERT, the length of the telomere gradually decreases (64). If the cells divide without telomeres, they would lose the end of their chromosomes that contain necessary information. Thus, the length of the telomere limits the lifespan of normal somatic cells (65). TERT activity has been found in most human cancers (66–69). Activation of telomerase was frequently (∼90%) found in HCC (70,71). The maintenance of telomere stability seems to be required for the immortalization of cancer cells. However, the mechanism by which telomerase is activated is not fully understood.

**Figure 1:**
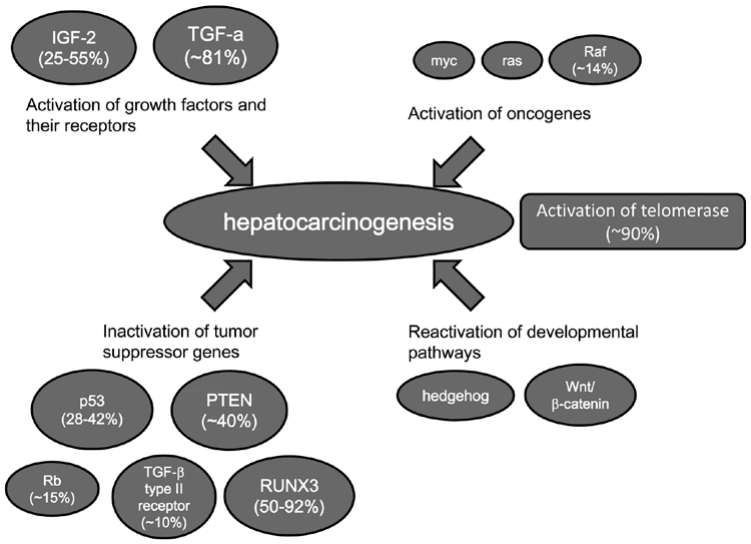
Gene alterations occurring in HCC. Gene alterations in human HCC are summarized; four major groups of genes are altered in HCC. Gene alterations of growth factors and tumor suppressor genes frequently occur in HCC.

Many cancer-related genes are altered in HCC. However, since the frequency of alteration for each individual gene is relatively low, the accumulation of alterations of cancer-related genes may be necessary for hepatocarcinogenesis. Hepatocarcinogenesis is tightly associated with chronic hepatitis or liver cirrhosis in which there are persistent inflammation and cell division of hepatocytes. Continuous inflammation induces oxidative DNA damage, and then DNA repair occurs occasionally accompanied with DNA misrepair, resulting in increased mutation frequency. Constant activation of cell division and the increased chances of DNA replication errors are important factors for the development of HCC (Fig. 1).

## 3. Experimental hepatocarcinogenesis *in vitro*

### Spontaneous immortalization

Normal human cultured cells are quite resistant to neoplastic transformation (72,73), whereas rodent cultured cells are transformed to neoplastic cells with relative ease. This species difference is probably due to the difficulty in immortalizing normal human cells *in vitro*, because human cells are strictly predestined to cellular aging. As far as we know, neither spontaneous immortalization nor spontaneous neoplastic transformation of normal human hepatocytes has been reported.

### DNA virus oncogenes

Oncogenic genes have been introduced in order to establish immortalized human hepatocytes. Various types of human cells can be immortalized with oncogenic genes from DNA viruses, such as simian virus 40 (SV40), adenovirus, and papillomavirus (74). Hepatocytes were successfully immortalized only by introducing the SV40 large T antigen (75); however, SV40 immortalization may have no relationship with hepatocarcinogenesis. Furthermore, there is no evidence that SV40 is related to human cancer (76).

### Retrovirus oncogenes

In studies of other types of cells, myc and ras were able to immortalize human fibroblasts and epithelial cells, respectively (77,78). These retroviral oncogenes are related to at least a few cases of HCC. However, there has been no report that these genes successfully immortalize human hepatocytes.

### HCV core protein

HCV core protein is known to induce oxidative stress, steatosis, and HCC in the patient with HCV (79). Ray *et al* introduced HCV core genomic region into primary human hepatocyte (80). Those cells became immortalized and exhibited continuous cell growth. Immortalization is necessary but not sufficient for hepatocarcinogenesis. However, HCV core protein transgenic mice developed HCC after the age of 16 months (81). Thus HCV core protein may relate to an important process in the multistep hepatocarcinogenesis.

### Chemical treatment and ionizing radiation

Although exposure to some chemical agents is closely related to human HCC, no successful malignant transformation of human hepatocytes by chemical agents or ionizing radiation *in vitro* has been reported.

### Characterization of immortalized human hepatocytes

Several human hepatocyte cell lines were established by transfection of SV40 T-antigen. THLE-2 and THLE-3 cells were established from an adult human liver autopsy sample (82). These cells were non-tumorigenic, but the population doubling levels of these cell lines were more than 100. These cell lines were established from liver epithelial cells, and they expressed albumin and cytokeratin 18 in early passages, thus suggesting that they expressed features of both hepatocytes and non-parenchymal cells. In a study of hepatocyte-specific functions of these cell lines, activities of the enzymes including cytochrome P-450 reductase, nicotinamide adenine dinucleotide phosphate, superoxide dismutase, catalase, glutathione S-transferase, and epoxide hydrolase were maintained.

Immortalized human hepatocytes were also established from surgically resected human adult liver by Schippers *et al*, using the SV40 T-antigen (75). These hepatocytes retained albumin secretion equivalent to that of normal primary human hepatocyte. Though there was no description of liver-specific enzymes, such as cytochrome P-450 reductase activity, their immortalized hepatocytes retained polarity, which is important for the formation of bile canaliculi. Interestingly, these hepatocytes maintained the multidrug-resistant P-glycoprotein, which is essential for the removal of toxic metabolites.

We established another immortalized human hepatocyte OUMS-29 from human embryonic liver using SV40 T-antigen (83). OUMS-29 cells have a population doubling level of more than 900. These cells produce liver-specific proteins, including albumin, transferrin, α-antitrypsin, and apolipo-protein A1. Furthermore, these cells also retain cytochrome P-450 reductase activity.

The study of *in vitro* carcinogenesis of human hepatocytes has shown that only the introduction of SV40 large T antigen can successfully immortalize cells. A major difficulty in the *in vitro* induction of carcinogenesis of human hepatocytes is the inadequacy of the available methods of culturing human hepatocytes. To solve this problem, methods of culturing human liver cells with hepatocyte characteristics need to be developed in the future.

The introduction of TERT might be a useful method for human hepatocyte carcinogenesis. TERT introduction into human primary hepatocytes increases the population doubling level, thus providing easy *in vitro* culture of primary hepatocytes.

Since telomerase activation is a common feature in HCC, telomerase activity may play a key role in hepatocarcinogenesis, especially in immortalization, because the immortalization of the cell is the initial step in the neoplastic transformation process. However, the cause and effect relationship between telomerase activation and hepatocarcinogenesis has not been elucidated yet.

## 4. Experimental animal model of hepatocarcinogenesis

Animal models of carcinogenesis play a critical role in understanding the mechanism of carcinogenesis. Many experimental hepatocarcinogenesis models have been developed (reviewed in ref. 84).

The H-ras or B-raf mutation was frequently found in rodent liver tumors (85,86); however, these mutations were infrequent in human HCC. The difference in gene alteration between rodent HCC and human HCC was also found in p53 mutations. Mouse HCCs generally lack p53 mutations, whereas this mutation is relatively frequent in human HCCs (18–50%) (87). This species difference needs to be considered in order to elucidate the molecular mechanism of human hepatocarcinogenesis. In spite of the species difference, animal models are still useful tools in understanding the process of development especially for the early stages of hepatocarcinogenesis.

## 5. Conclusion

**Figure 2:**
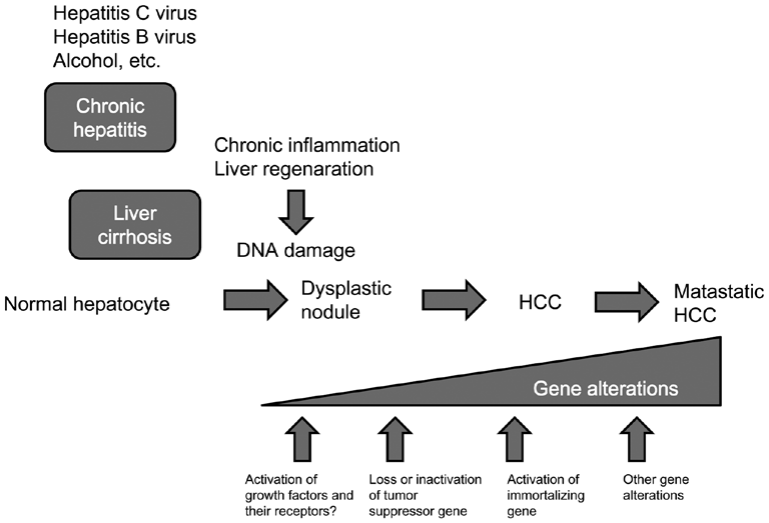
Sequential gene alterations in the human liver leading to HCC. Chronologic sequence of the development of human HCC and gene alterations; HCC develops in the setting of chronic inflammation due to viral hepatitis or alcoholic liver injury. Hepatocarcinogenesis may begin in dysplastic nodules consisting of pre-neoplastic hepatocytes. Accumulation of gene alterations in dysplastic hepatocytes leads to HCC. Further gene alterations are responsible for the malignant transformation of HCC.

As in the case of other types of human cancers, hepatocarcino-genesis seems to be a multistep process in which multiple cancer-related genes are altered. These genetic changes are related to tumor suppressor genes, oncogenes, reactivation of developmental pathways, and growth factors and their receptors. Although numerous genes are altered in HCC, the frequency of each individual gene alteration is relatively low. Telomerase activation is the common feature of HCC and is closely related to immortalization. Thus, telomerase activation may be the common effect of cancer-related genes (Fig. 2).

Neoplastic transformation of human hepatocytes has not yet been achieved in an *in vitro* model of human hepatocarcinogenesis. Normal human cells are quite resistant to neoplastic transformation. Although several human immortalized hepatocyte cell lines have been established, they have been immortalized only by introducing with the SV40 large T antigen. Given that immortalization is only an initial step of the neoplastic transformation process, immortalized human hepatocytes can become useful tools for the elucidation of hepatocarcinogenesis, especially for the initial step of multistep hepatocarcinogenesis.

## Abbreviations:

HCC: hepatocellular carcinoma;
HBV: hepatitis B virus;
HCV: hepatitis C virus;
Rb: retinoblastoma;
LOH: loss of heterozygoty;
PTEN: phosphatase and tensin homolog;
RUNX3: runt-related transcription factor 3;
APC: adenomatous polyposis coli;
PTCH: patched;
SMO: smoothened;
TGF-α: transforming growth factor-α;
IGF-2: insulin-like growth factor-2;
TGF-β: transforming growth factor-β;
TERT: telomerase reverse transcriptase;
SV40: simian virus 40
